# 

**Published:** 1916-05-27

**Authors:** 


### CONTENTS.

| | PAGE |
|---|---|
| **The Leprosy Case** ... ... ... ... ... ... | 181 |
| **Shall We Sweat the Crippled Soldier?** ... | 182 |
| **Hospital and Institutional News** ... ... ... | 183 |

The Failure of the Red Cross Conference—The Henry Saxon Snell Prize—Medical Practitioners and the Military Service Bill—Scholarships for Women Medical Students—The British Hospital, Port Said—The Princess Louise Hospital—The Lighter Side of Looting—The Proposed South Shields Hospital in Sunnyside Lane—Elizabeth Fry's Training School—A Curious Point in Scottish Law—Parish Councils and Tuberculosis Work—Wales's New Sanatorium—A Point for Education Committees—The Day of Peace Offerings—A Fly Bibliography—The Asylum Workers' Association—The Editorials in War Hospital Magazines—The Lighter Side of the War Hospital—Infant and Maternal Welfare Work in Liverpool—Extra Health Visiting at Manchester—School Inspection in Devon—The Fruits of Research in the Dustbin—Panel Chemists and their Remuneration—Vagrancy in Surrey—The Inspection of Massage Establishments and Lying-in Homes—The Courage of Dublin Hospital Staffs—Increased Patients in Infirmaries—Flat Rates in Poor-Law Infirmaries—Hospital Representative for the Pharmaceutical Council.

**Military Amputations** ... ... ... ... ... 189

I.—A Reversion to Pre-Listerian Methods.

**On Acute Nephritis in Children** ... ... ... 190
The Importance of Urinalysis in Diseases of the Young.

**Human Temperaments** ... ... ... ... ... 191

III.—The Temperament of the Artist.

**The Leicester Royal Infirmary** ... ... ... 193

**The School Doctor** ... ... ... ... ... 193
The School Doctor and the Mentally Defective Child.

**The Matrons' and Sisters' Department** ... 195
State Registration in Great Britain.
Round the Hospitals.
"A Tilt in the Courts"—Medical Students and Nurses—"National Egg Scandal"—Some Suggestions for the College—A Tribute to V.A.D.s—The Model Store for Matrons.

**Textile Supplies for Hospitals** ... ... ... 197

XII.—The Purchasing of Stores.

**The Editor's Letter-Box** ... ... ... ... 199
Work for the College of Nursing?—Withholding the Annual Report: An Explanation Asked For—The Bishop's Last.

**From Across the Seas** ... ... ... ... 200
The Ideal Hospital Superintendent—A Record Quotation.

**Matrons' and Sisters' Appointments** ... ... 200
**The National Medical Union** ... ... ... 200
**Passing Events and Topics** ... ... ... ... 200
**Annual Meetings** ... ... ... ... ... 200
**The Institutional Worker** ... ... (Suppl.) 1
War Work at the Voluntary Hospitals.
What Northampton General Hospital has done.
Questions Invited.
Enquiries and Answers.

